# Dissemination and Implementation Research: Intersection between Nursing Science and Health Care Delivery

**DOI:** 10.1155/2013/802767

**Published:** 2013-09-19

**Authors:** Deborah Vincent, Marie Hastings-Tolsma, Kathleen R. Stevens

**Affiliations:** ^1^University of Arizona, College of Nursing, Tucson, AZ 85721, USA; ^2^Division of Women, Children, and Family Health Nursing, College of Nursing, University of Colorado Denver, Aurora, Denver, CO 80204, USA; ^3^Improvement Science Research Network, University of Texas Health Science Center San Antonio, San Antonio, TX 78229, USA

Dissemination is the targeted distribution of information and intervention materials to spread knowledge and evidence-based interventions to a larger audience (NIH, 2011). Implementation is the use of strategies to promote the systematic uptake of evidence-based practices into routine practice and healthcare policy to improve the quality and effectiveness of health services (IOM, 2012). The dissemination and implementation (D&I) lexicon includes many terms like translational research, knowledge translation, knowledge exchange, improvement science, and technology transfer. Dissemination and implementation researchers and practitioners must carefully consider strategies for translating evidence into practice to optimize health care delivery. D&I science focuses on translating knowledge into practice by facilitating stakeholder access to findings using a variety of strategies and designs that include pragmatic trials, evidence-based quality improvement, health technology assessment, systematic reviews, randomized clinical trials, and comparative effectiveness research. This collection of articles begins with broad-level perspectives such as priorities, collaboration, and IRB regulations and moves to specific examples of D&I research.

Given the tremendous possibilities for D&I research, where should a nation or organization focus its resources? The manuscript by K. Stevens and J. Ovretveit addresses developing national improvement priorities for D&I research to guide researchers and funders. Results of this study suggest that, at least for the United States, the most important and urgent needs include care coordination, implementation of evidence-based practice, improving structure and clinical processes, and developing a culture of quality and safety. These types of research studies require transdisciplinary teams and an infrastructure that supports academic-practice partnerships. The article by F. Puga, K. Stevens, and D. Patel identifies best practices in developing a healthcare improvement research network that integrates various theories, methods, and perspectives of each discipline into a cohesive unit able to focus on complex research issues and topics. A companion article by D. Patel, K. Stevens, and F. Puga identifies variation in institutional review board approval process for D&I studies especially when these studies focus on quality improvement initiatives across multiple sites.

Two manuscripts describe specific efforts to implement evidence-based practices. The manuscript by S. Daniels et al., describes the efforts of one Veterans Administration hospital to implement screening for dysphagia in patients who presented to the emergency department with symptoms of stroke. Multidisciplinary team cooperation and organizational support were major facilitators of this quality improvement study, while lack of adequate training perceived lack of time was seen as barriers to implementation. The manuscript by A. Wallace et al. describes the process and framework used to integrate diabetes self-management support into routine diabetes care provided in four community health clinics. 

The final manuscript by Y. Ogbolu et al. assessed the level of nurse compliance with evidence-based guidelines for preventing mother-to-child transmission of HIV in a resource-limited country. Although making up the greatest portion of the health care workface in most countries, educational and scope of practice barriers often hinder their effectiveness in improving health outcomes. 

By compiling these papers, we hope to enrich our readers with respect to the need for and variety of D&I studies. We also hope to stimulate thought for future studies to enhance the translation of evidence into practice and to improve health outcomes for populations. 

*Deborah Vincent* *Deborah Vincent* *Marie Hastings-Tolsma* *Marie Hastings-Tolsma* *Kathleen R. Stevens* *Kathleen R. Stevens*

